# Reconstructing Local Population Dynamics in Noisy Metapopulations—The Role of Random Catastrophes and Allee Effects

**DOI:** 10.1371/journal.pone.0110049

**Published:** 2014-10-31

**Authors:** Edmund M. Hart, Leticia Avilés

**Affiliations:** Department of Zoology, University of British Columbia, Vancouver, British Columbia, Canada; Arizona State University, United States of America

## Abstract

Reconstructing the dynamics of populations is complicated by the different types of stochasticity experienced by populations, in particular if some forms of stochasticity introduce bias in parameter estimation in addition to error. Identification of systematic biases is critical when determining whether the intrinsic dynamics of populations are stable or unstable and whether or not populations exhibit an Allee effect, i.e., a minimum size below which deterministic extinction should follow. Using a simulation model that allows for Allee effects and a range of intrinsic dynamics, we investigated how three types of stochasticity—demographic, environmental, and random catastrophes— affect our ability to reconstruct the intrinsic dynamics of populations. Demographic stochasticity aside, which is only problematic in small populations, we find that environmental stochasticity—positive and negative environmental fluctuations—caused increased error in parameter estimation, but bias was rarely problematic, except at the highest levels of noise. Random catastrophes, events causing large-scale mortality and likely to be more common than usually recognized, caused immediate bias in parameter estimates, in particular when Allee effects were large. In the latter case, population stability was predicted when endogenous dynamics were actually unstable and the minimum viable population size was overestimated in populations with small or non-existent Allee effects. Catastrophes also generally increased extinction risk, in particular when endogenous Allee effects were large. We propose a method for identifying data points likely resulting from catastrophic events when such events have not been recorded. Using social spider colonies (*Anelosimus spp.*) as models for populations, we show that after known or suspected catastrophes are accounted for, reconstructed growth parameters are consistent with intrinsic dynamical instability and substantial Allee effects. Our results are applicable to metapopulation or time series data and are relevant for predicting extinction in conservation applications or the management of invasive species.

## Introduction

Understanding how different kinds of noise affect our ability to reconstruct the intrinsic dynamics of populations is important for both theoretical [1]–[3] and practical [4]–[6] applications, in particular if some types of stochasticity introduce, systematic biases in parameter estimation in addition to error [7]. Accurate reconstructions are important from a theoretical perspective for assessing how often and under what conditions populations exhibit intrinsic dynamical instability [8]–[10], or whether, and of what magnitude, populations exhibit Allee effects. The possibility that parameters that lead to intrinsic dynamical instability may be present in populations has been controversial, as boom and bust patterns of growth are expected to be unsustainable over long periods [10]–[13]. Recognizing the presence and magnitude of Allee effects is important as these effects cause populations to exhibit a minimum size below which deterministic extinction should follow [14]–[16]. Allee effects arise when populations of very small sizes have negative growth due to processes such as difficulty in locating mates [17], pollen limitation [18], mating system [19], or minimum group size for social species [14], [20]. In interaction with intrinsically and extrinsically driven population size oscillations, Allee effects should thus increase a population's risk of extinction [21]. Accurate characterization of population stability and Allee effect sizes is also important in applications ranging from the preservation of endangered species [22] to the management of invasive species [23]. Angulo et al [22], for instance, found that populations of the endangered island fox in the Channel islands were in danger of going extinct due to a predator driven Allee effect, and that only with the inclusion of Allee effects in their models could they explain the steep decline in population size. Tobin et al [24] have also found that managers can implement eradication plans that exploit Allee effects in invasive species. Likewise, slowing down the spread of invasive species may require identifying possible source populations, which, due to fast growing and perhaps unstable local dynamics, may be a frequent source of propagules colonizing sink habitats [32], [56]. It is therefore critical from conservation and management perspectives that the presence of intrinsic dynamical instability and Allee effects be recognized and accurately characterized. This in turn requires that we identify the effects of different forms of stochasticity on our ability to reconstruct the endogenous dynamics of populations.

Three kinds of stochasticity may affect the dynamics of populations: demographic stochasticity, environmental stochasticity, and random catastrophes [25]. *Demographic stochasticity* affects small populations and is caused by the chance realization of individual probabilities of death and reproduction, which are typically Poisson processes [26]. *Environmental stochasticity* is variation in an extrinsic factor (biotic or abiotic) that affects the birth and death rates of all individuals in a population across all age classes [25] and is important regardless of population size. *Random catastrophes* are large disturbances leading to negative growth via large mortality events [25], [27]–[29]. Both demographic and environmental stochasticity are likely to introduce error in the estimation of population parameters because they cause fluctuations randomly in a positive and negative direction. Catastrophes, on the other hand, may cause systematic biases in population parameter estimation as their effects are unidirectional [30]. Recognizing the occurrence and frequency of catastrophic events is thus important from both theoretical [25], [28], [31], [32] and practical [27], [29], [33], [34] standpoints, as catastrophes may increase a population's extinction probability, influence effective population sizes, and potentially alter metapopulation dynamics [35], [36]. Furthermore, catastrophes may be particularly problematic for populations subject to Allee effects as these events may cause populations to fall below the lower equilibrium point and thus go extinct [37], [38].

We explore the effects of these three kinds of stochasticity on population parameter estimation using a mix of simulations and actual data. The simulation model allows for Allee effects and represents the growth of local populations in a metapopulation sampled at two time steps, but our results are also applicable for time series data. The parameters used for the simulations are loosely based on the biology of social spiders, organisms in which intrinsic benefits of group-living bind individuals together in relatively isolated local populations (called "colonies"), which, through processes of local extinction and recolonization, form part of a metapopulation [39]. Because social spiders occupy environments in which persistence may not be possible in the absence of cooperation [40], [41], their colonies exhibit Allee effects [40], [42].

Population growth functions with an Allee effect exhibit three equilibria as the functions intersect the 45° identity line (i.e., when *N_t+1_*  =  *N_t_*) at three points—the trivial, N = 0, lower equilibrium point; an intermediate unstable equilibrium; and an upper stable equilibrium. Populations that fall below the intermediate unstable equilibrium are expected to eventually map down to zero, while those remaining above this point should converge to or oscillate around the upper stable equilibrium (see Avilés [14], for solutions for the specific model used here). The slope of the growth function around the upper stable equilibrium (hereafter ‘slope’) in a discrete time series model is a quantity that defines whether a population is stable (>−1) or unstable (<−1) [43], [44], while the intermediate unstable equilibrium (hereafter IUE) is a measure of the strength of an Allee effect.

Our simulation results show that random catastrophes, unlike other forms of noise, cause systematic bias in parameter estimation, characterizing local populations as dynamically stable when local dynamics were actually deterministically unstable, in particular when Allee effects were strong. When Allee effects were small or non-existent, on the other hand, random catastrophes lead to an overestimation of Allee effect size. As we suspect that random catastrophes are a more common form of noise in natural populations than currently recognized, we caution that unless catastrophic events are recognized and accounted for, accurate functional reconstruction of population dynamics is nearly impossible. We also show that random catastrophes, in particular in interaction with strong Allee effects, greatly increase a population's extinction probability. We illustrate these findings using as models for populations field colonies of two social spider species of the genus *Anelosimus*. For colonies of one of the species catastrophic events had been documented and could thus be accounted for, while, for the other, we show that without knowledge of catastrophes estimates of population growth parameters can be nonsensical (e.g., predict a population with zero as its only expected equilibrium). Investigators interested in estimating any facet of population dynamics (stability, Allee effects, etc…) thus need to recognize whether or not the system experiences catastrophic events and which data points are likely affected. Using the patterns of catastrophe-generated points in the phase space in our actual data and simulations, we classified unknown points as affected or not by a catastrophe using a generalized boosting regression tree model [45]. We used simulation data to generate a large training dataset to produce this model, which we then applied to simulation and actual data with known catastrophes. After accounting for known or suspected catastrophes in our social spider data we show that colony growth parameters for the two species are consistent with the presence of Allee effects and endogenously unstable colony dynamics.

## Methods

### Overview

The social spiders after which our simulations are modeled form colonies that represent self-sustaining local populations that grow, proliferate and become extinct without mixing with one another and that are subject to both positive and negative density dependence [39], [40]. Local populations in our simulated system are thus independent of one another (i.e., do not exchange migrants [39]) and subject to an Allee effect due to benefits of group living and cooperation [14]. Such population structure is similar to organisms that colonize relatively isolated local patches and have within patch social facilitation leading to an Allee effect (e.g. [46]). The conclusions of the models, however, are not restricted to species with a metapopulation structure, but should be applicable to any species with populations whose dynamics may be affected by catastrophes and Allee effects.

### Local population growth

Because the particular way in which different models regulate density dependence may produce idiosyncratic results [47], [48], we considered two quite different functions for the growth of local populations—a version of the Ricker model [49] with an added positive density dependent factor that creates an Allee effect due to cooperation (for details, see Avilés [14]), and a version of the Hassell model [50], where an Allee effect is introduced to reflect declining fitness in smaller populations [51].

For the Ricker model we used the version by Avilés [14], as follows:
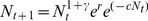
(1)


Where 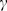, assumed to range between 0 and 1, introduces an Allee effect by causing some components of fitness to increase as *N* increases, so that fitness is maximum at intermediate population sizes (see Avilés [14] for full details); *r* is the intrinsic population growth rate; and *c* determines the strength of negative density dependence.

For the Hassell model, we used the Fowler and Ruxton [51] version, as follows 
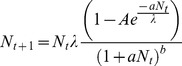
(2)


In this model *A* and *γ* control the size of the Allee effect, *λ* is the maximum fecundity of an individual, *a* is the strength of density dependence and *b* is the strength of competition.

Simulation procedures were otherwise identical for both models, as described below and in the appendix (Appendix S1). As we obtained qualitatively very similar results with the two models, we discuss the results of the Ricker model, which is more appropriate for our social spider example, here, and give results from the Hassell model in the appendix (Appendix S1).

### Metapopulation dynamics

We used a lattice map [44], [52] to create our metapopulation system. Each cell within the lattice represented a local population, which at every time step grew according to one of the two equations above. All populations shared the same global parameter values and rules for colonization, extinction, and dispersal. The dynamics of the local populations were independent of one another, because only one population could exist in a cell and individuals did not move between cells (thusly colonies). Cells made available to colonization by local extinction events, however, could be colonized by individuals derived from any other local population in an island model of patch colonization. Dispersal was density-dependent, a common feature of both vertebrate [53] and invertebrate [54] populations. Our implementation, described fully in the appendix (Equation S1 and S2 in Appendix S1), sets the probability of dispersal increasing as a population exceeds the size at which its growth becomes negative (Equation S1 in Appendix S1) and, for simplicity, colonization is independent of distance to empty cells [55]. Extinction occurred when populations were mapped below their IUE as a result of noise, often combined with very fast growth leading to wide population size oscillations

### Simulation steps

We ran each simulation for 100 time steps (each step representing a generation) with a metapopulation size of 150 local patches. Every time step had the following order of operations: local population growth, potential catastrophic events, dispersal, and recolonization. During each time step we applied demographic and environmental stochasticity and random catastrophes to each local population. Demographic stochasticity was in the form of random draws from a Poisson distribution for the number of surviving offspring. Environmental stochasticity was applied by drawing random values of *r* and *c*, in eq. 1, or *λ* and *a*, in eq. 2, from a gamma distribution. A gamma distribution was used because it allowed us to draw real numbers that were always greater than zero. Finally, based on data from one of our social spider species, *Anelosimus domingo* Levi we assumed that local populations were hit by a catastrophic event with probability 0.25 per generation [42]. Such catastrophic events led to sudden and drastic reductions in population size of a magnitude that, based again on quantitative field estimates, we assumed to be a decreasing function of the original population size (see Appendix S1 for implementation).

### Experimental design and analysis

We examined two different population sizes and Allee effect strengths, with their sizes roughly based on actual data from our two social spider species—the already mentioned, *A. domingo*, which has relatively small colonies (local populations) and is suspected to have strong Allee effects and a larger-bodied species, *Anelosimus eximius* Keyserling, which has relatively large colonies and weak Allee effects (see ‘Fitting actual data’ below, for details) [40]. A small population had a maximum size of approximately 150 individuals, and a large population was approximately 1500 individuals. Allee effect sizes were set as a ratio of the IUE of eqs. 1 [14] or 2 to the maximum population size. In weak Allee effect populations the ratio was 0.5%, and 10% for strong Allee effects. We thus created four different population scenarios and simulated five different levels of environmental stochasticity crossed with six different catastrophe levels (we show only a subset of these in the graphs to facilitate interpretation). The levels of environmental stochasticity were varied by the coefficient of variation (CV) of the parameter distributions (for *r* and *c*, in eq. 1, or *λ* and *a* in eq. 2), from 0 (demographic stochasticity only) to 0.22 in 5 equal increments (see Appendix S1 for details). By using the CV we had a dimensionless measure of variation that we could apply across all of our population parameters. We varied the magnitude of the catastrophes by setting the fraction of a population that would be lost at *N* = 0 (i.e., the intercept of Equation S4, Figure S1 in Appendix S1) to one of six levels, ranging from 50% loss, for mild catastrophes, equally spaced up to 90% loss, for extreme catastrophes, as well as a scenario with no catastrophes. These corresponded to an average minimum population loss ranging from 45% to 85%, for our small populations (∼150 individuals), and from 10% to 45%, for our large populations (∼1500 individuals) (Figure S1 in Appendix S1).

### Simulation and parameter estimation

We ran 1000 simulations for each of 120 parameter combinations for each of the two models (Ricker or Hassell, eq. 1 and eq. 2, respectively). After each simulation, which was run for 100 time steps, we estimated parameters by bootstrap resampling with 1000 replicates, estimating all parameters for eq. 1 or eq. 2 via non-linear least squares regression (in R with the *nls()* function), a method that assumes additive normal errors. While our simulations used a variety of error structures to represent various biological processes (Poisson, for offspring production; gamma, for environmental stochasticity; binomial for the probability of catastrophes, etc., see Appendix S1), additive normal errors were used for curve fitting to emulate a method commonly used for parameter estimation from actual data. Upon analyzing the residuals of the nonlinear model fit to the simulation data we found them to be symmetrical around the mean, regardless of whether or not catastrophes were part of the error structure. We thus do not expect that the mismatch between the simulated error structures and the normal error assumption of the reconstructions will differentially affect model outputs with and without catastrophes. Each parameter estimate was then used to calculate the slope of the functions around the upper stable equilibrium [43], [44] and the mean intermediate unstable equilibrium (IUE). These were the two parameters of interest since they are biologically meaningful quantities derived from the more abstract parameters of eq. 1 or 2.

We recorded the mean and standard deviation of each bootstrap resulting in a data set of 1000 simulated means of each 1000 bootstrapped estimates for each model. Based on those 1000 means we calculated, based on Yu et al [56], the mean normalized factor bias (*B*_MNF_) and the mean normalized absolute error (*E*_MNAF_) for the slope and the IUE, as follows:
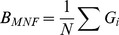
(3)

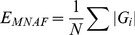
(4)

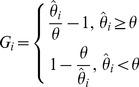
(5)where 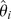 is the bootstrap average of simulation *i* out of *N* simulations and *θ* is the known true value that the simulations were based on. Bias is a measure of how far off in one direction an estimate is from its true value, and error denotes how wrong an estimate is without considering direction. Negative bias means that the estimator is biased towards underestimating a parameter value and positive, vice-versa, with values close to zero indicating no bias. The normalized estimators of [56] take into account the sometimes asymmetrical properties of bias and error metrics. However, the inverse is true if *θ* is negative. In that case, more positive values mean 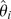 is more negative, and if 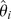 is becoming positive, bias will become more negative.

### Fitting actual data

We have data on the change in size from one generation to the next of naturally occurring colonies of our two neotropical social spiders, *A. eximius* and *A. domingo*. Both species occur in tropical rainforest habitats in northern South America [39]. Colonies of *A. eximius* can be found in the forest understory or along forest edges, with colonies at the latter habitat potentially growing into the tens of thousands. *A. domingo*, in contrast, is only found in the forest understory where it forms colonies that may contain up to a few thousand spiders. In addition to growing through internal recruitment without mixing with one another, colonies of both species also give rise to dispersal events at large colony sizes, thus producing “daughter” colonies [39]. Colonies may also go extinct without leaving descendant colonies. The nests of both species consist of clear silk structures built surrounding portions of live vegetation. These structures provide clear enough visibility of their contents so that demographic changes can be tracked through time. We thus recorded at two-week intervals, for a period of 10 months, the contents of 39 *A. domingo* colonies at the Jatun Sacha Biological Station (S 1.07° W 77.61°, Napo Province, Ecuador) [42]. This allowed us to determine their change in size (number of adult and subadult females and of egg sacs) for two consecutive generations and to detect the occurrence of random catastrophes, defined as sudden (within a 2-week period) losses of >30% of a colony's population associated with an independently documented external event, such as a falling branch or heavy rainfall that caused web destruction. Given a strong correlation between nest and colony size [41], in the case of *A. eximius*, we inferred from the dimensions of the nests the size at consecutive generations of 103 river-edge colonies at the Cuyabeno Nature Reserve (S 0.03", W 76.2" –76.3", Sucumbíos Province, Ecuador). In this case we did not observe the colonies at sufficiently short time intervals to document the occurrence of catastrophic events.

Using non-linear least squares we fit eq.1 to both data sets. In the case of *A. domingo*, we fit both the full data set and just the colonies that didn't suffer catastrophic events. For *A. eximius* we fit the same equation to the full data set, and to a subset of the data from which colonies suspected to have been subject to catastrophes were excluded. We classified unknown points as having been affected or not by a catastrophe using a generalized boosting regression tree model [45], which we developed using a large training data set produced with our simulation model. The training data set consisted of 2000 simulations of small populations with weak Allee effects, and intermediate environmental noise and catastrophe levels of 60–90%. We chose small populations with weak Allee effects because this combination gave the most accurate predictions across a range of other simulation test data sets (12–20% misclassification). To account for effects of scale, we standardized all measurements by dividing them by the 99^th^ percentile value of population size so all population sizes were on the same scale. Due to this scaling we had to exclude two extreme outlier points from the *A. eximius* data set ([7654, 0] and [16637,0]) as, otherwise, our stochastic classifier algorithm performed very poorly. We tested our regression tree model against data with known catastrophes from an additional set of simulations and our *A. domingo* data set. We then applied the method to our *A. eximius* data set to infer which data points are likely to reflect the effect of catastrophic events. Additionally, using the *A. domingo* patterns as a guide and without prior knowledge of the stochastic classifier results, we visually assessed which *A. eximius* data points most likely represented catastrophes. We present the results of both reconstructions.

## Results

**Figure 1:**
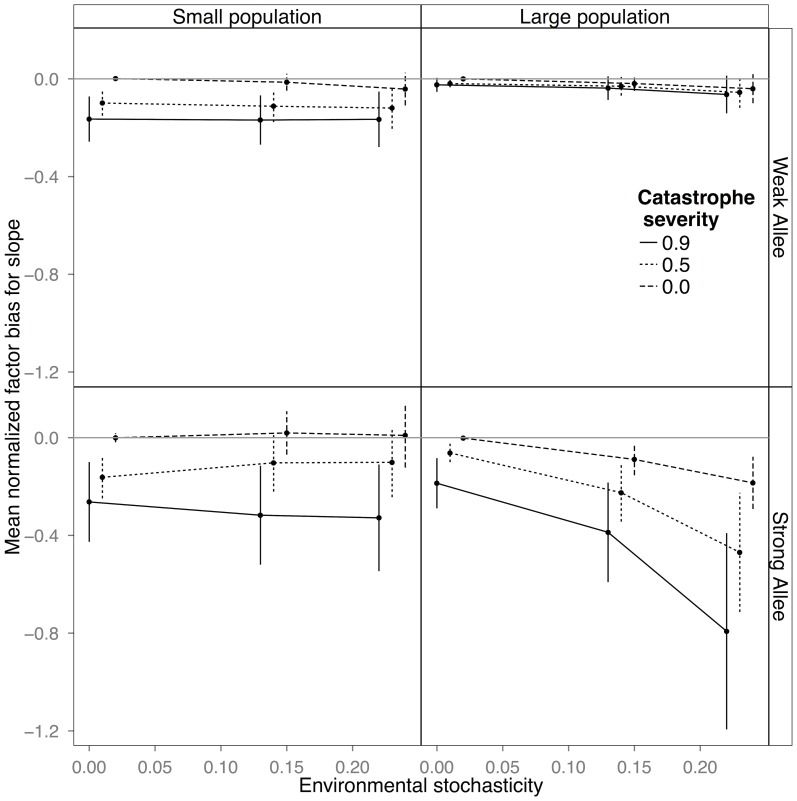
Plots of mean normal factor bias and error in estimates of the slope of the growth function at the identity line in small populations with a weak Allee effect, large populations with a weak Allee effect, small populations with a strong Allee effect, and large populations with a strong Allee effect. The magnitude of the bias (eq. 4) can be seen in the distance of the estimate from the 0.0 line, with a bias >0.0 indicating that the parameter was more negative than the true value and a bias <0.0, that it was more positive than the true value (i.e., in the latter case, the slope was flatter than its actual value), this is because the true value in this case is negative. Vertical bars represent error on the bias estimate (eq. 5). Environmental stochasticity increases along the x-axis. Bias was always greatest in populations with a strong Allee effect.

**Figure 2:**
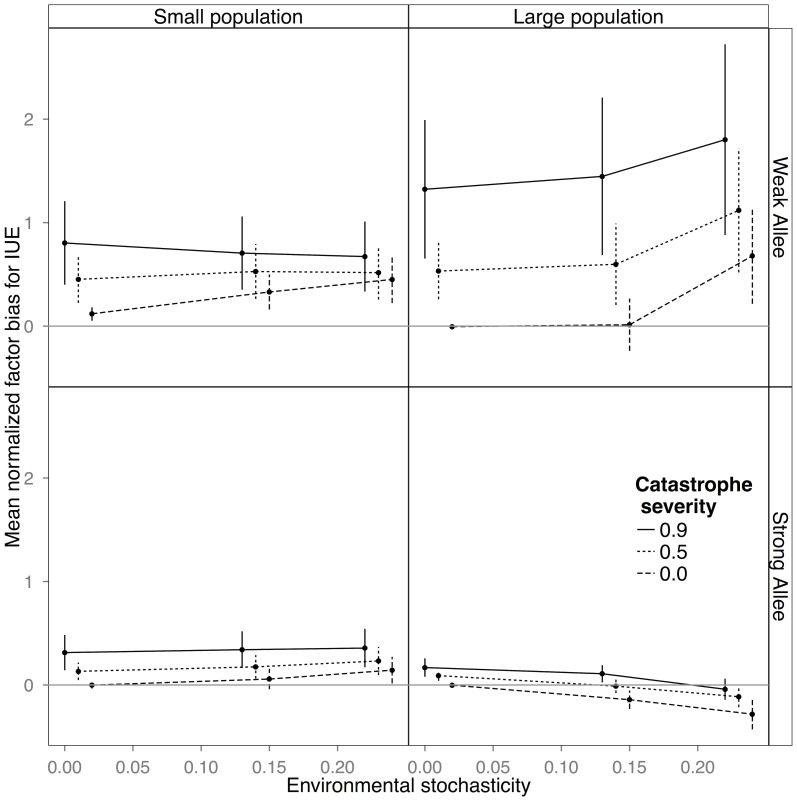
Plots of mean normal factor bias and error in estimates of the intermediate unstable equilibrium (IUE) in small populations with a weak Allee effect, large populations with a weak Allee effect, small populations with a strong Allee effect, and large populations with a strong Allee effect. The magnitude of bias (eq. 4) can be seen in the distance of the estimate from the 0.0 line, with a bias >0.0 indicating that the magnitude of the parameter was underestimated and a bias >0.0, that it was overestimated, with the vertical bars representing error (eq. 5). Environmental stochasticity increases along the x-axis. Although in large populations bias (overestimation) was greatest with weak Allee effects, considering that the bias metric is normalized by its true value, in absolute numbers, overestimation could represent fewer individuals in the small Allee effect case than in the strong Allee effect one (see text for a numerical example and potential ramifications).

Demographic stochasticity alone introduced no error or bias to the estimates of the slope and led only to a slight overestimation of the IUE at the smaller population size with weak Allee effects (Figures 1 and 2, long-dashed line in each panel, corresponding to 0 catastrophes, and zero environmental stochasticity). Added environmental stochasticity, in the absence of catastrophes, resulted in increasingly less precise estimates as the level of noise increased (Figure 1 and 2, long-dashed line), but introduced no bias to the slope or IUE estimates (Figure 1 and 2, vertical bars on long-dashed lines) under most circumstances. The exception was at the highest levels of environmental stochasticity when there was an interaction with Allee effect size such that the slope parameter was estimated to be flatter when Allee effects were large, and the IUE was overestimated with small Allee effects (Figure 2). Catastrophes (short-dashed and solid lines), on the other hand, always led to the estimation of a less steep slope parameter (i.e. a slope value closer to 0 than the true value), thus predicting more stable dynamics than actually present (Figure 1). Catastrophes also led to over-estimation of the IUE, which in relative terms was more dramatic for populations with small Allee effects (Figure 2). Generally errors caused by catastrophes were large even for low levels of environmental stochasticity (Figures 1 and 2), indicating that catastrophes introduced both bias and error, while other forms of noise, with some exceptions, introduced mostly error.

The largest differences in bias were between populations with weak and strong Allee effects, rather than between small and large populations. While the bias always increased with increasing catastrophe severity, its magnitude was dependent on both the parameter being measured and the underlying Allee effect size. The bias of the slope was magnified in populations with strong Allee effects (Figure 1), while the bias in the IUE was greatest in populations with weak Allee effects (Figure 2). In large populations with strong Allee effects there was an interaction between environmental stochasticity and catastrophes, with the negative bias in the slope increasing nonlinearly with the size of the catastrophes and the intensity of noise (Figure 1).

**Figure 3:**
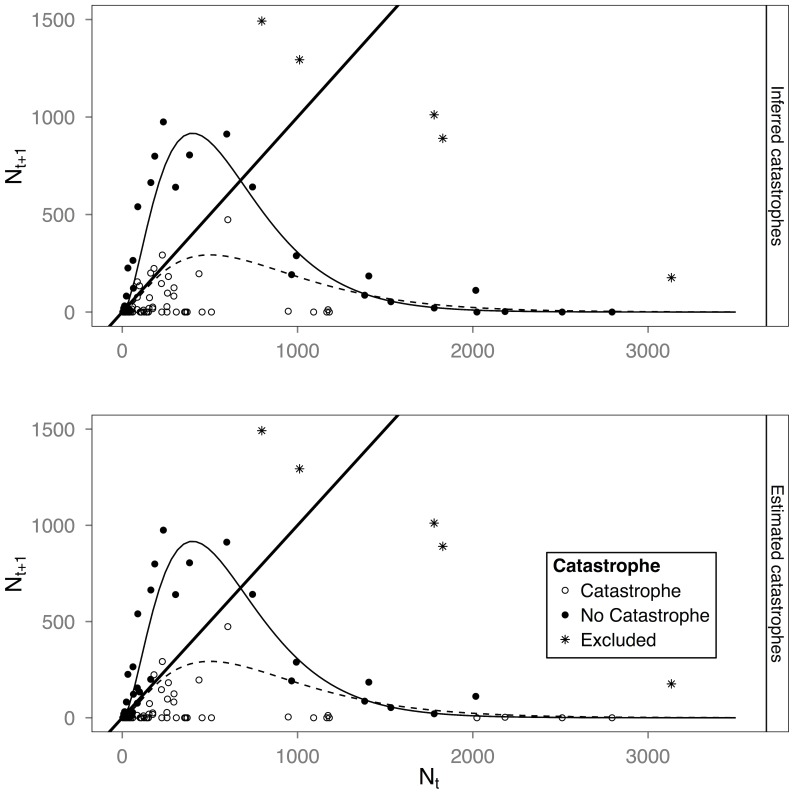
Reconstruction of the function governing the growth of colonies of the neotropical social spider *A. eximius* given two methods to assign points to possible catastrophic events: visual assessment, using the *A. domingo* pattern (Figure 4) as a guide, and using a boosting regression tree method (see Methods for details). Dashed lines are a best fit line using eq. 1 and fitting all the data; solid lines are the fit excluding points suspected to have been affected by catastrophes. The graphs illustrate how lacking knowledge of catastrophes can lead to the nonsensical inference of a population that cannot exist, while after suspected catastrophes are removed the inference of intrinsic dynamical instability (i.e., a slope steeper than −1) is supported. In both cases, the presence of an Allee effect is detected. Inferred values after suspected catastrophes are removed: IUE: 10 (1.07, 97.58) (95% C.I.); slope: −1.33 (−0.51, −1.92); and in estimated catastrophes from random forests IUE: 21 (4.82, 117.21); slope: −1.29 (−0.46, −1.81). Note that the figures do not show two data points with extreme values on the x-axis (([7654, 0] and [16637,0]); these points, however, were included in the analyses. The five colonies shown with an asterix and excluded from the analyses underwent a proliferation event in the transition between generations, thus belonging to a different dynamical regime.

**Figure 4:**
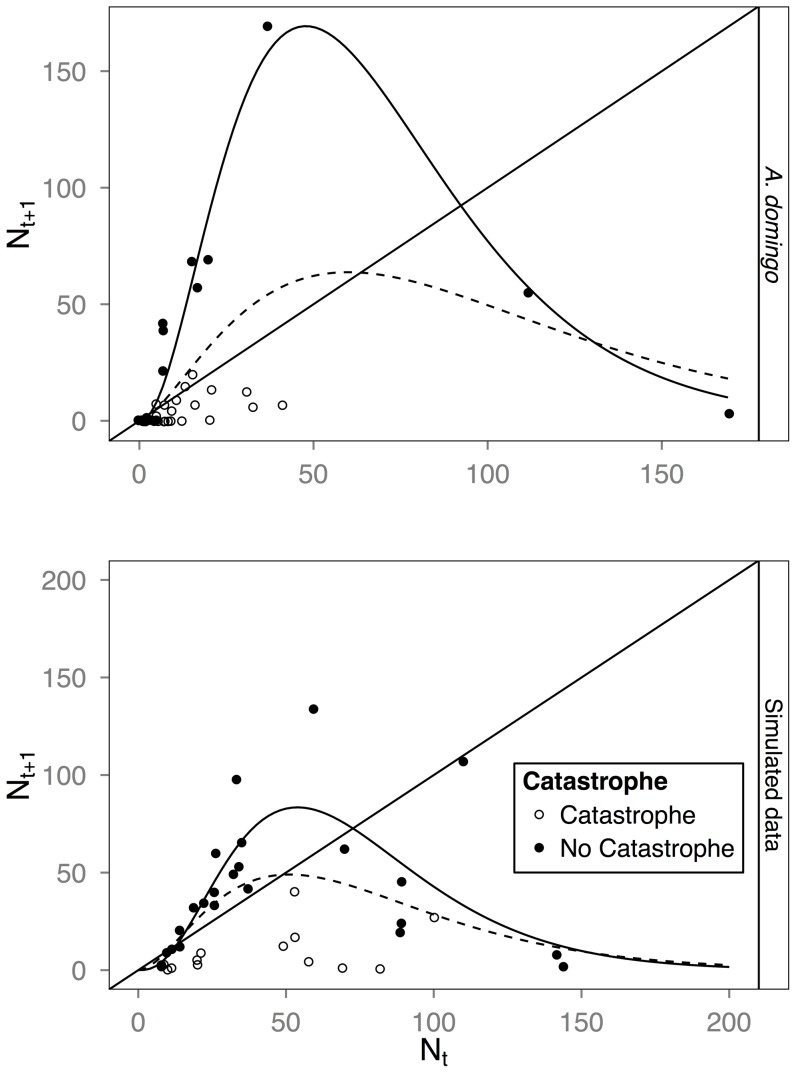
Reconstruction of the function governing the growth of colonies of the neotropical social spider *A. domingo* and one set of simulated data. In both, data points affected by catastrophic events were known. Dashed lines are a best fit line using eq. 1 and fitting all the data; solid lines are the fit excluding points known to have been affected by catastrophes. Both graphs show a much less steep curve when catastrophes are included, thus predicting stable endogenous dynamics, when they actually are not. Inferred values for *A. domingo* when catastrophes were included (dashed line): IUE: 4.75 (1.32, 69.73) (95% C.I.), slope: −0.14 (−2.83, 2.54); when catastrophes were removed (solid line): IUE: 3 (1.16, 7.12), slope: −1.5 (−2.74, −1.13).

In both our actual and simulated data it is clear that the slope estimate when catastrophes are included (slope at the intersection of the dashed and identity lines, Figures 3 and 4) is much flatter than when catastrophes are removed (slope corresponding to the solid line). When we fit all the data for *A. eximius*, the function does not intersect the identity line (Figures 3, dashed lines), indicating that our model fit predicts a population that cannot exist. Removing points suspected to reflect the effect of catastrophes (Figure 3, solid lines), however, reveals a significant Allee effect and a slope steeper than −1 (see legend Figure 3, for inferred IUE and slope values). Our fits of the *A. domingo* data including documented catastrophes estimate a larger IUE and a flatter slope than when we removed colonies subject to catastrophes (Figure 4, dashed and solid lines, respectively). Our analyses thus reveal that colonies of both species exhibit an Allee effect and may be subject to strong intrinsic dynamical instability. They are also consistent with our predictions of catastrophes causing an underestimation of the slope and an overestimation of the IUE, the latter especially so when the Allee effect is small. A similar pattern is obtained in our simulated data (Figure 4). Note that the boosted regression tree model, which we used to infer points likely affected by catastrophes in the *A. eximius* data set, had a misclassification rate of 32% when applied to the *A. domingo* data set and 12–20%, when applied to the simulation data sets. Any inferences derived from this model, therefore, should be considered tentative.

**Figure 5:**
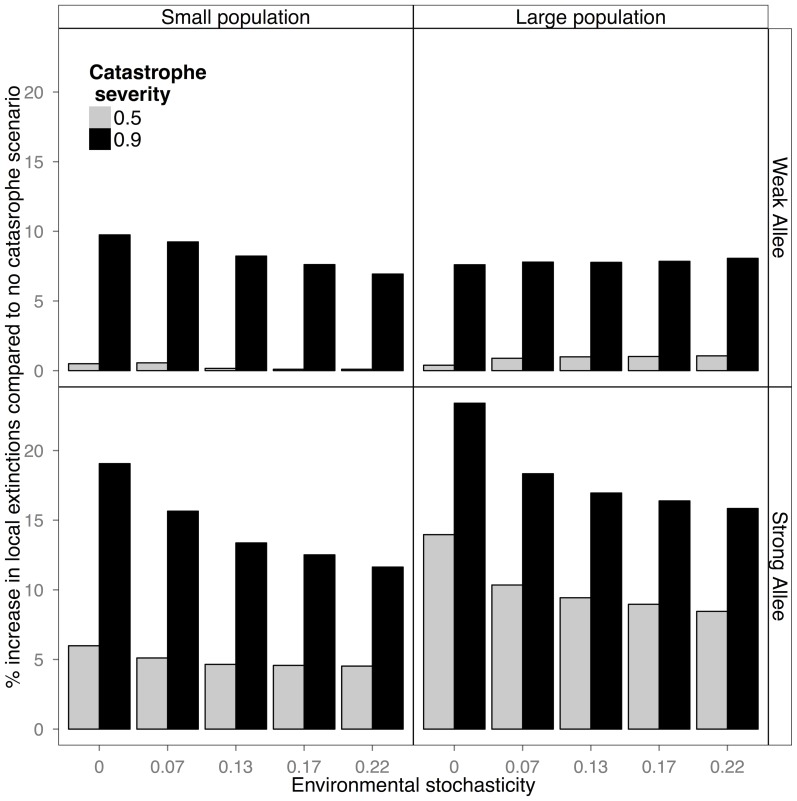
The percent increase in local extinctions for our smallest (0.5) and highest (0.9) catastrophe levels, over a scenario with no catastrophes. Generally this decreases with increasing environmental noise because the background extinction rate increases with increasing noise. Both small and large populations with small Allee effects have almost no difference at the 0.5 catastrophe level, whereas high Allee effect populations show an increase in extinction rate with either catastrophe level.

Finally, the presence of random catastrophes interacted with Allee effect size to determine the extinction probability of the local populations. Thus, the probability of a local population going extinct was greatly increased, relative to a no catastrophe scenario, when catastrophe severity and Allee effect size were large (Figure 5).

## Discussion

Catastrophic events introduced a systematic bias in our estimates of stability and, given a weak Allee effect, in our estimates of the intermediate unstable equilibrium, IUE. Catastrophes tended to result in reconstructions with flatter curves (i.e., less steep slopes; Figures 3 and 4) and larger IUEs. Other forms of stochasticity (environmental, demographic), on the other hand, tended to introduce only error (Figures S2–S5 in Appendix S1). Catastrophes also caused large amounts of error, regardless of the degree of environmental stochasticity, greater than even the most extreme levels of noise without any catastrophes we considered. As catastrophes may not be a rare occurrence—our data on the neotropical social spider *A. domingo*, for instance, suggest that 30% of its colonies may be affected by catastrophes in any one generation ([42] and L. Avilés and P. Salazar, unpublished data)—this form of noise should therefore be an important consideration when assessing population stability or Allee effect size, more so than even high levels of environmental stochasticity. After correcting for catastrophes, our reconstruction of *A. domingo* colony dynamics had a smaller IUE and a more negative slope, indicative of growth parameters that should yield intrinsic dynamical instability (Figure 4).

Perhaps the most important finding of our simulations was the interaction between underlying Allee effect strength and catastrophes in biasing parameter estimates. In populations with a strong Allee effect there was a large bias in estimation of the slope, with estimated parameter values predicting much more stable dynamics than actually present, while in populations with a weak Allee effect there was a large bias in estimating the IUE. Bias in the slope in populations with strong Allee effects is most likely due to the sensitivity of those populations to local extinction, as even weak catastrophes resulted in a high probability of local extinction when Allee effects were strong (Figure 5). The increase in local extinction then magnified the noisy signal already introduced by catastrophes, creating extremely biased estimates. Populations with a weak Allee effect, on the other hand, were robust to local extinction, except at the most extreme catastrophe levels (Figure 5). The large bias in the IUE estimate in populations with a weak Allee effect (Figure 2) likely reflected the fact that the bias metric is normalized by its true value, so that, in absolute terms, overestimation in number of individuals could be greater for the strong Allee effect case. Nonetheless, in large populations the amount of overestimation in a weak Allee effect case could be significant, even in absolute terms. Thus, a 400% bias in the weak Allee effect case could mean estimating an IUE of 30 individuals when the actual number was 6 (400% bias * 6), a discrepancy potentially more serious than, in a strong Allee effect situation, estimating an IUE of 144 individuals when the actual number was 90 (assuming a 0.6% overestimation), especially in situations involving endangered species or invasive species management that rely on Allee effect estimates. These results thus highlight that estimating small Allee effects with any precision may be difficult. Nonetheless, with truly small Allee effect sizes, even a relatively large bias may not be of great practical concern given unavoidable measurement error.

Even though both Allee effects and stochasticity due to catastrophes have received much theoretical attention [15], [25], [28], [31], [57], the interaction between the two has rarely been studied. One exception is the study of Groom [18] where she was able to detect an Allee effect by measuring pollination success and extinction as a function of isolation and patch size in populations of an annual plant. In her study catastrophes were the number one cause of extinction. Had she not recorded the occurrence of such events, she would have been unable to parse the effects of environmental stochasticity and Allee effects.

Detection of catastrophes associated with either time-series data from a single population or data on multiple local populations in a metapopulation requires that data be collected at relatively short time intervals. With sparsely collected data, the cause of significant drops in local population size is otherwise impossible to gauge. Thus for *A. domingo*, where a colony generation takes about four months (L. Avilés, unpublished data), our data collection involved bi-weekly censuses of the size and age structure of the colonies. This allowed the detection of sudden declines in population size that could be associated with direct or indirect evidence of catastrophic events, such as strong rains or falling branches that damaged the web. Detection of catastrophic events, on the other hand, was not possible for our *A. eximius* data set where colony size data was recorded only once every generation. Once data points known or suspected to have been subject to catastrophes in the *A. domingo* and *A. eximius* data sets, respectively, were removed, we were able to estimate parameter values that were consistent with the presence of an Allee effect and of intrinsically driven population instability, both reasonable inferences for our social spider species [14], [20]. These inferences would not have been possible without recognizing the possibility of catastrophic events affecting colony growth patterns in our study populations, (Figures 3 and 4).

Accurate estimates of population stability and Allee effect sizes are important for predicting extinction in conservation applications for both preserving rare species [38], [58] and managing invasive species [24]. Knowing the size of an Allee effect alters the approach to conservation because Allee effects create a useful alternative to minimum viable population size [21], [58], [59]. An overestimation of IUE's could alter conservation priorities if species are thought to be close to their unstable equilibria when in fact they are far away from them. If managers are aware of the possibility and effect of catastrophes, they can successfully integrate them to estimate parameters for management purposes [33]. Fast growing, albeit locally unstable populations in a metapopulation system can serve as a source for colonizing propagules [35] of sink populations. This may facilitate the spread of invasive species into sink habitats where survivorship of just a few propagules is low [60], but fast growing and unstable source populations send out a large number of migrants. Managers may also want to change eradication strategies depending on the complexity (stability) of the underlying dynamics of a population [61]. Accurate estimation of the magnitude of an Allee effect is perhaps even more important for invasive species management [62] because it has been shown to have a large impact in how invasive species spread [23], [24], [63], [64].

Accurate estimation of the slope parameter and underlying endogenous dynamics is also important from a theoretical perspective. The possibility that intrinsic dynamical instability and chaos may characterize the dynamics of natural populations has been recognized since the early 70s [43], [65]. Empirical studies, however, have suggested that most populations lay in the region of stability [9], [10], a finding that, in light of our results, may need to be revised if parameter estimates for some of those populations had been biased due to unaccounted for random catastrophes. Thus, endogenous parameters that would lead to intrinsic dynamical instability may be more common than currently appreciated assuming natural populations are affected by events that lead to drastic reductions in their population size.

### Limitations of the study, open questions, and future work

Our inferences are based on the Ricker and Hassell models and may not hold if another model is better fitting to data. Furthermore it remains unclear how factors such as lags, greater than first order feedbacks, or age structure would influence our conclusions. Even though we had not documented directly the occurrence of catastrophes in the *A. eximius* data set, by analyzing the patterns arising from the simulations and in the data set where catastrophes were documented, we have developed a preliminary method to infer after the fact what data points may be affected by this type of noise. This method will need to be refined before it can be used more generally, such as by using other classification schemes (e.g., support vector machines) and assessing performance against other datasets.

## Conclusions

In summary, we found that catastrophes cause both bias and error in estimating both the slope and Allee effect sizes (the IUE) from population data. The direction of this bias is always to estimate a less steep slope and a larger IUE than their actual value. Environmental stochasticity alone increased error in parameter estimation, but rarely introduced bias. The magnitude of this bias, however, varied with the underlying population's Allee effect size rather than the population size itself. We also observed that populations with strong Allee effects had especially high extinction rates even with small catastrophes. Given the importance of population stability and the prevalence of Allee effects [17], [58], it is thus essential that investigators record the occurrence of catastrophes so that unbiased estimates of these two parameters are obtained. When catastrophes are recognized, they can either be removed from the dataset to estimate population parameters, or taken into account with alternative model structures.

## Supporting Information

Appendix S1 (PDF)
