# Supplementary material for: Reconstructing Local Population Dynamics in Noisy Metapopulations—The Role of Random Catastrophes and Allee Effects
Source: PLoS One. 2014 Oct 31;9(10):e110049. doi: 10.1371/journal.pone.0110049 (PMC4216000; doi:10.1371/journal.pone.0110049)
Supplement: Appendix S1 — (PDF) [file pone.0110049.s001.pdf]

Reconstructing local population dynamics in noisy metapopulations—the role of random catastrophes and Allee effects

Edmund M. Hart and Leticia Avilés

Appendix

Model details

We carried out a complete set of simulations with Allee-effect versions of the Ricker or Hassell population growth models (see Avilés [1] and Fowler & Ruxton [2], respectively, for details on the particular versions used). We used a lattice map to simulate our metapopulation. Local populations grew largely independently from one another, consistent with the metapopulation structure and dynamics of social spider species, which constitute the empirical models for our simulations. In these species colonies are self-sustaining local populations with migration between them being low to nonexistent [3].Instead *colonization* occurred whenever a local site became empty. Each site was recolonized by drawing a random variate from a Poisson distribution with a fixed lambda that was 10% greater than the calculated intermediate unstable equilibrium point (IUE; see main text Introduction for details). *Extinction* occurred when a population was mapped below this IUE as a result of noise, often coupled with extreme instrinsic dynamical instability and was not, otherwise, controlled in the simulations.

Dispersal was density dependent. The dispersal probability of a local population increased as its size grew above the stable equilibrium point. We call this point N_{max}. For the Avilés [1] version of the Ricker model, this point is defined by

$$N_{\max} = \frac{(1+\gamma)}{c} \qquad \text{S1}$$

where γ is a parameter that causes some components of fitness to increase as N increases, thus creating an Allee effect and resulting in fitness being maximum at intermediate population sizes (see Aviles [1] for full details), and *c* determines the strength of negative density dependence. For the Hassell model we used numeric root finding methods to find $N_{max.}$ in R with the rootSolve package.

If $N_{ti} > N_{\max}$ then the dispersal probability of a local population was $p = 1 - N_{\max} / N_{ti}$. The number of dispersers, *D*, were modeled as a binomial process proportional to the size of the inequality, as follows:

$$D_{ti} \sim \text{Bin}\left(N = N_{ti} - N_{\max}, p = 1 - \frac{N_{\max}}{N_{ti}} \right) \qquad \text{S2}$$

Demographic stochasticity: All populations in the simulation underwent demographic stochasticity, as follows. For the Ricker function:

$$N_{t+1} = \sum_{j=0}^{j=N_t{}^{1+\gamma} \mathrm{e}^{(-cN_t)}} \text{Pois}(\mathrm{e}^{r}) \qquad \text{S3}$$

For the Hassell equation:

$$N_{t+1} = \sum_{j=0}^{j=N_t \frac{\left(1-A\mathrm{e}^{\frac{-aN_t}{\gamma}}\right)}{(1+aN_t)^b}} \text{Pois}(\lambda) \qquad \text{S4}$$

These are simply eqs. 1 or 2, where one of its terms is treated as a Poisson process.

Environmental stochasticity: To simulate local environmental stochasticity, each population at each time step was given a random value from a gamma distribution for the growth and carrying capacity parameters, *r* and *c* (or λ and *a* for the Hassell model). The shape and rate parameters of the gamma distribution varied with the amount of environmental stochasticity we simulated. These levels were determined by increasing the coefficient of variation (C.V.) on each modified parameter (*r, c,* λ*, a*) from 0 (demographic stochasticity only) up to a C.V. = 0.22

Catastrophes: We used a fixed binomial probability that a local population would be hit by a catastrophe (set in the simulations at 0.25 or roughly 25% of the local populations going extinct each generation) based on the catastrophe probability estimated from data on one of our social spider species, *A. domingo*. This is a binomial form of the Poisson approach used by Lande (1993). The fraction of individuals surviving in affected populations was drawn from a binomial with probability:

$$N_{ti} \sim \mathrm{Bin}\left(N = N_{ti}, p = 1 - \left[\frac{1}{\mathrm{e}^{(\beta_1 + \beta_2 * N_{ti} + \varepsilon)}}\right]\right) \qquad \text{S5}$$

Based on our empirical data [4], in (S5) the fraction of individuals lost to the population (the second factor of the function) is assumed to be an exponentially declining function of population size, with β_1 being the intercept; β_2, the rate of decline, and ε, a normally distributed error term. Parameter values were chosen based on the *A. domingo* data. The catastrophe levels are variations in the intercept of the function, β_1 (Fig. S1).

Hassell model results and discussion: We observed very similar patterns in our Hassell model simulations to those of the Ricker model simulations. In the absence of catastrophe, estimates of both the slope and the IUE in populations subject to increasing environmental stochasticity showed increasing error, but little bias (Figure S6, S7, long dash lines). When catastrophes occurred, there was an increase in both error and bias regardless of environmental stochasticity. This pattern is the same as we saw in our results with the Ricker model where catastrophes overwhelmed any effect of other forms of stochasticity. Slope estimation was most biased in populations with a strong Allee effect (Figure S6C, S6D), similar to our results from the Ricker simulations. However we found that small populations with strong Allee effects were the most biased under the Hassell model, which differs from the Ricker simulations where we saw the greatest amounts of bias in large populations with strong Allee effects. The highest levels of bias for the IUE were observed in small populations regardless of the strength of Allee effect (Figure S7A, S7C), with small populations and weak Allee effects having the highest bias. Large populations had the same amount of bias regardless of Allee effect size (Figure S7B, S7D). This is different from the results we observed in the Ricker model where bias was greatest in weak vs. strong Allee effect size, not population size.

While some differences exist between the Ricker and Hassell model, we observed otherwise very similar overall patterns in both simulations. Catastrophes across all levels of environmental stochasticity created strong bias and error. Furthermore the extent to which bias and error were present in a population with catastrophic events interacted with

the underlying population parameters. In both the Ricker and Hassell simulations we found that the amount of bias introduced by a given catastrophe size varied with either population size or Allee effect size. The similar overall patterns obtained in both models, despite their very different underlying assumptions (see main text) argue for the generality of our results concerning the effects of different forms of stochasticity, and in particular random catastrophes, on the estimation of endogenous dynamics and Allee effect size in populations.

1. Avilés L (1999) Cooperation and non-linear dynamics : An ecological perspective on the evolution of sociality. Evol Ecol Res 1: 459–477.

2. Fowler M, Ruxton G (2002) Population dynamic consequences of allee effects. J Theor Biol 215: 39–46.

3. Avilés L (1997) Causes and consequences of cooperation and permanent-sociality in spiders. In: Choe J, Crespi B, editors. The evolution of social behaviour in insects and arachnids. Cambridge University Press. pp. 476–498.

4. Aviles L, Salazar P Unpublished data.

Table 1.

	Small population	Large population
Weak Allee effect	$r = 1$, $c = 0.0567$, $\gamma = 0.9$	$r = 0.27$, $c = 0.005$, $\gamma = 0.8$
Strong Allee effect	$r = 0.007$, $c = 0.0567$, $\gamma = 0.23$	$r = 0.0002$, $c = 0.00567$,<br>$\gamma = 2$

Table 1: Parameter values for Ricker model simulations

Table 2.

	Small population	Large population
Weak Allee effect	λ = 70 , a = 0.01, b = 6, A= 1, γ = 1	λ = 80 , a = 0.001, b = 5.5 , A= 1, γ = 1
Strong Allee effect	λ = 90 , a = 0.001, b = 18 , A= 1, γ = 1	λ = 100 , a = 0.0001, b = 20 , A= 1, γ = 1

Table 2: Parameter values for Hassell model simulations

Figure Legends

Figure S1. The fraction of the population lost at different catastrophe levels. This is a function of existing population size such that larger populations lose a smaller fraction of their overall population. These values are term *p* in eq. S4.

Figure S2. Histograms of 1000 bootstrapped estimates of the slope from the Ricker equation simulations, at 3 different levels of environmental stochasticity, 0, 0.13, and 0.22, and three different catastrophe levels in small populations. Vertical lines represent the true value.

Figure S3. Histograms of 1000 bootstrapped estimates of the slope from the Hassell equation simulations, at 3 different levels of environmental stochasticity, 0, 0.13, and 0.22, and three different catastrophe levels in large populations. Vertical lines represent the true value.

Figure S4. Histograms of 1000 bootstrapped estimates of the IUE from the Ricker equation simulations, at 3 different levels of environmental stochasticity, 0, 0.13, and 0.22, and three different catastrophe levels in small populations. Vertical lines represent the true value.

Figure S5. Histograms of 1000 bootstrapped estimates of the IUE from the Hassell equation simulations, at 3 different levels of environmental stochasticity, 0, 0.13, and

0.22, and three different catastrophe levels in large populations. Vertical lines represent the true value.

Figure S6. Plots of mean normal factor bias and error in estimates of the slope of the growth function at the identity line for models based on the Hassell equation (eq. S5) in (A) small populations with a weak Allee effect, (B) large populations with a weak Allee effect, (C) small populations with a strong Allee effect, and (D) large populations with a strong Allee effect. The magnitude of the bias (eq. 4) can be seen in the distance of the estimate from the 0.0 line, with the vertical bars representing error (eq. 5). Environmental stochasticity increases along the x-axis. Bias was always greatest in populations with a strong Allee effect.

Figure S7. Plots of mean normal factor bias and error in estimates of the intermediate unstable equilibrium point (IUE) in models based on the Hassell equation (eq. 2) (A) small populations with a weak Allee effect, (B) large populations with a weak Allee effect, (C) small populations with a strong Allee effect, and (D) large populations with a strong Allee effect. The magnitude of bias (eq. 4) can be seen in the distance of the estimate from the 0.0 line, with the vertical bars representing error (eq. 5). Environmental stochasticity increases along the x-axis. Bias was always greatest in small populations; there was relatively no difference between weak and strong Allee effects in large populations.

Figure S1.

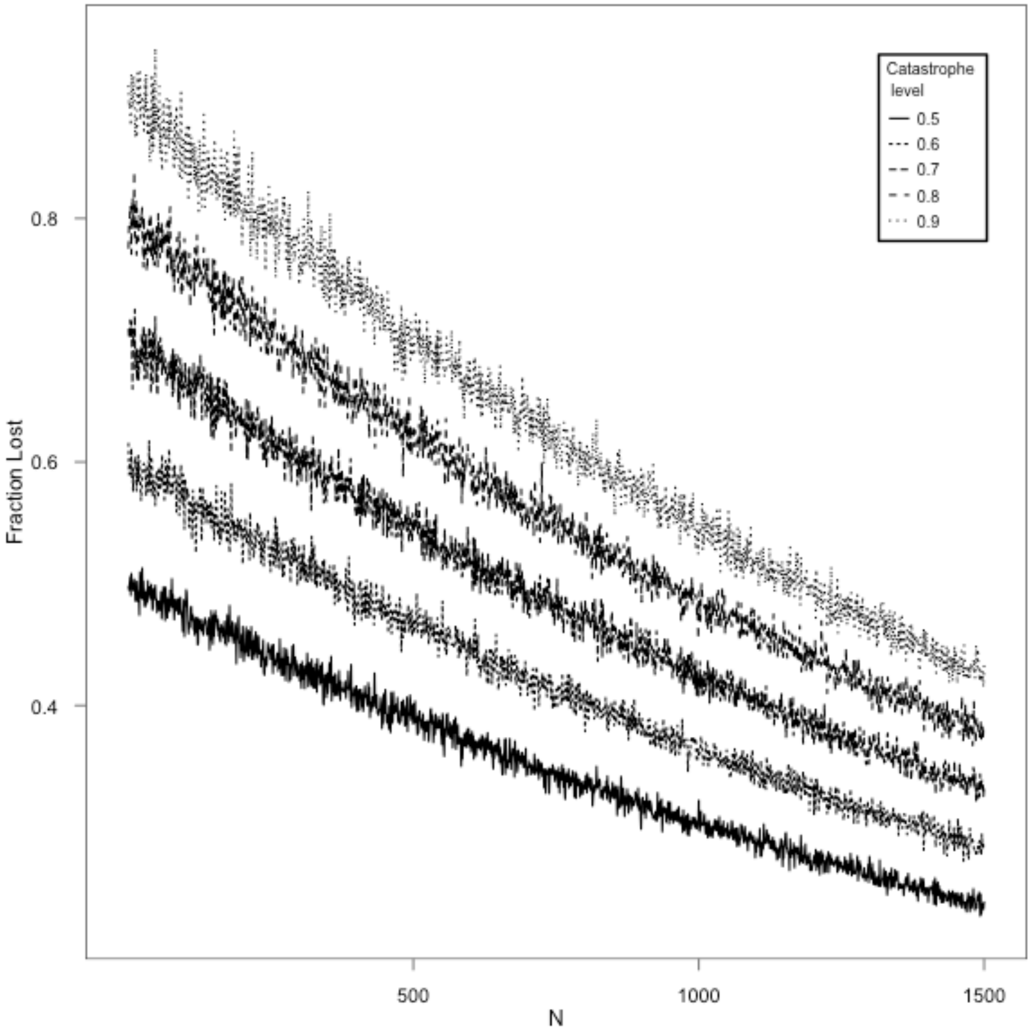
Catastrophe level
0.5
0.6
0.7
0.8
0.9
Fraction Lost
0.8
0.6
0.4
500
1000
1500
N

Figure S2

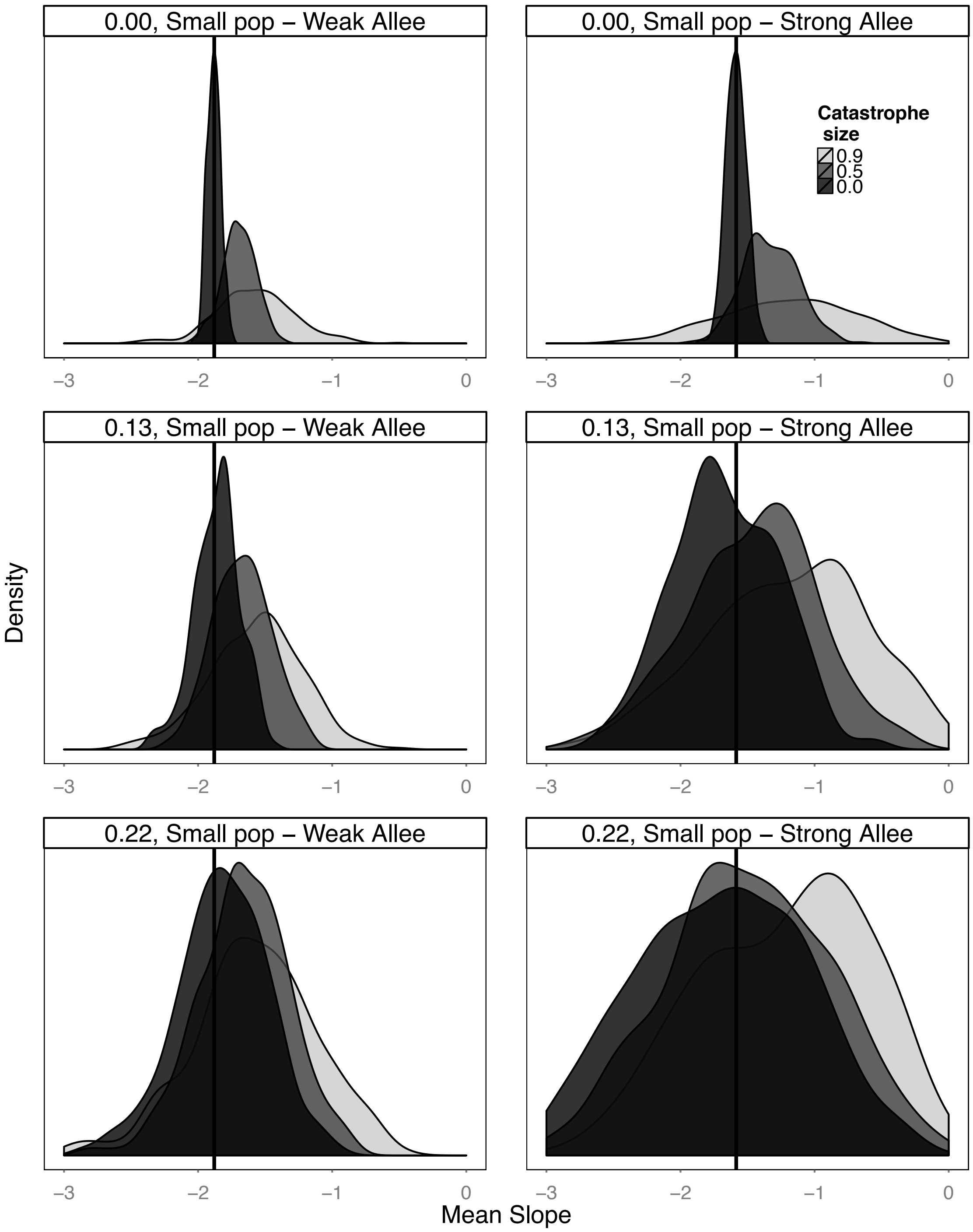
0.00, Small pop – Weak Allee
0.00, Small pop – Strong Allee
0.13, Small pop – Weak Allee
0.13, Small pop – Strong Allee
0.22, Small pop – Weak Allee
0.22, Small pop – Strong Allee
Catastrophe size
0.9
0.5
0.0
Density
Mean Slope
–3
–2
–1
0

Figure S3

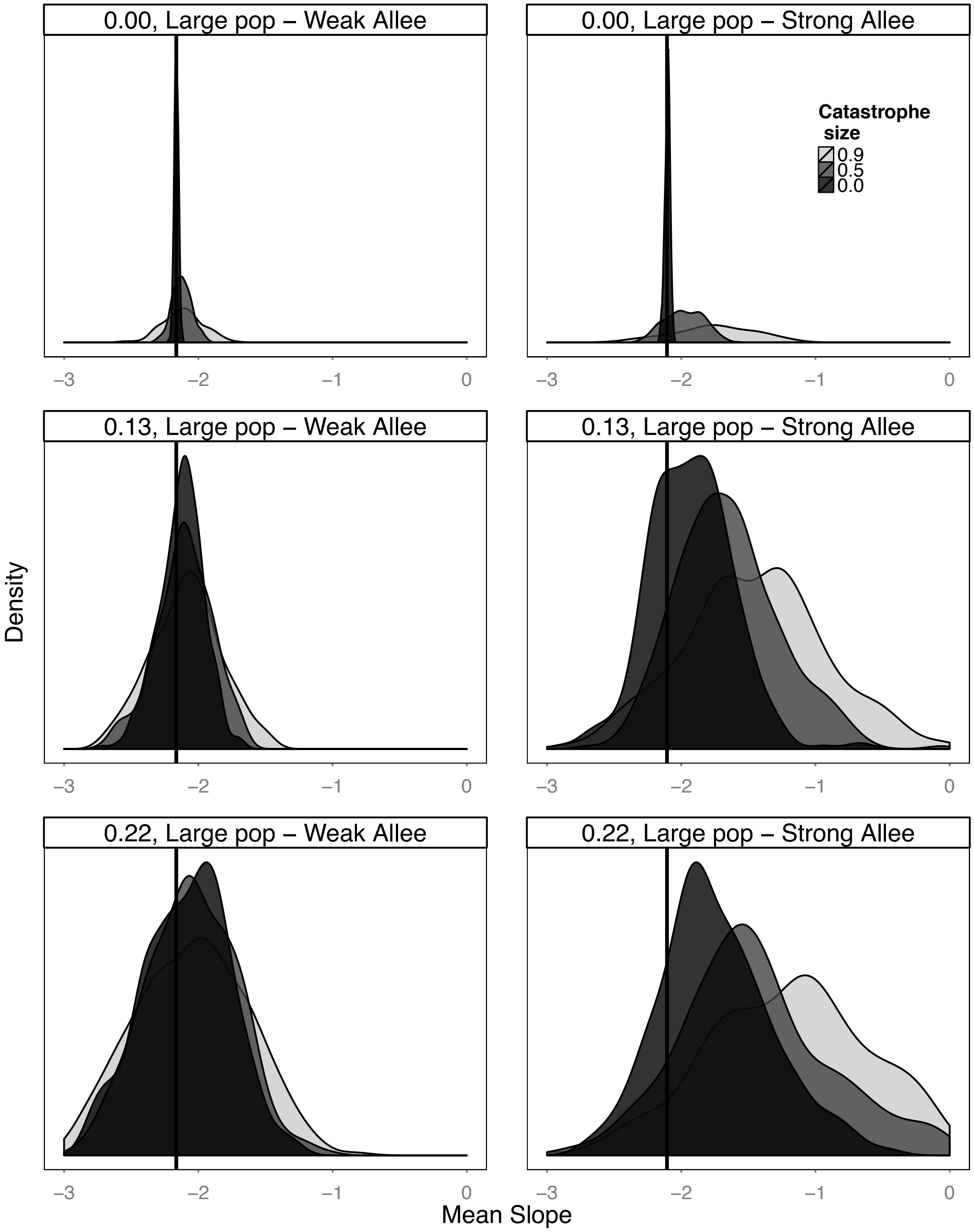

0.00, Large pop – Weak Allee
0.00, Large pop – Strong Allee
Catastrophe size
0.9
0.5
0.0
0.13, Large pop – Weak Allee
0.13, Large pop – Strong Allee
0.22, Large pop – Weak Allee
0.22, Large pop – Strong Allee
Density
–3 –2 –1 0
Mean Slope

Figure S4

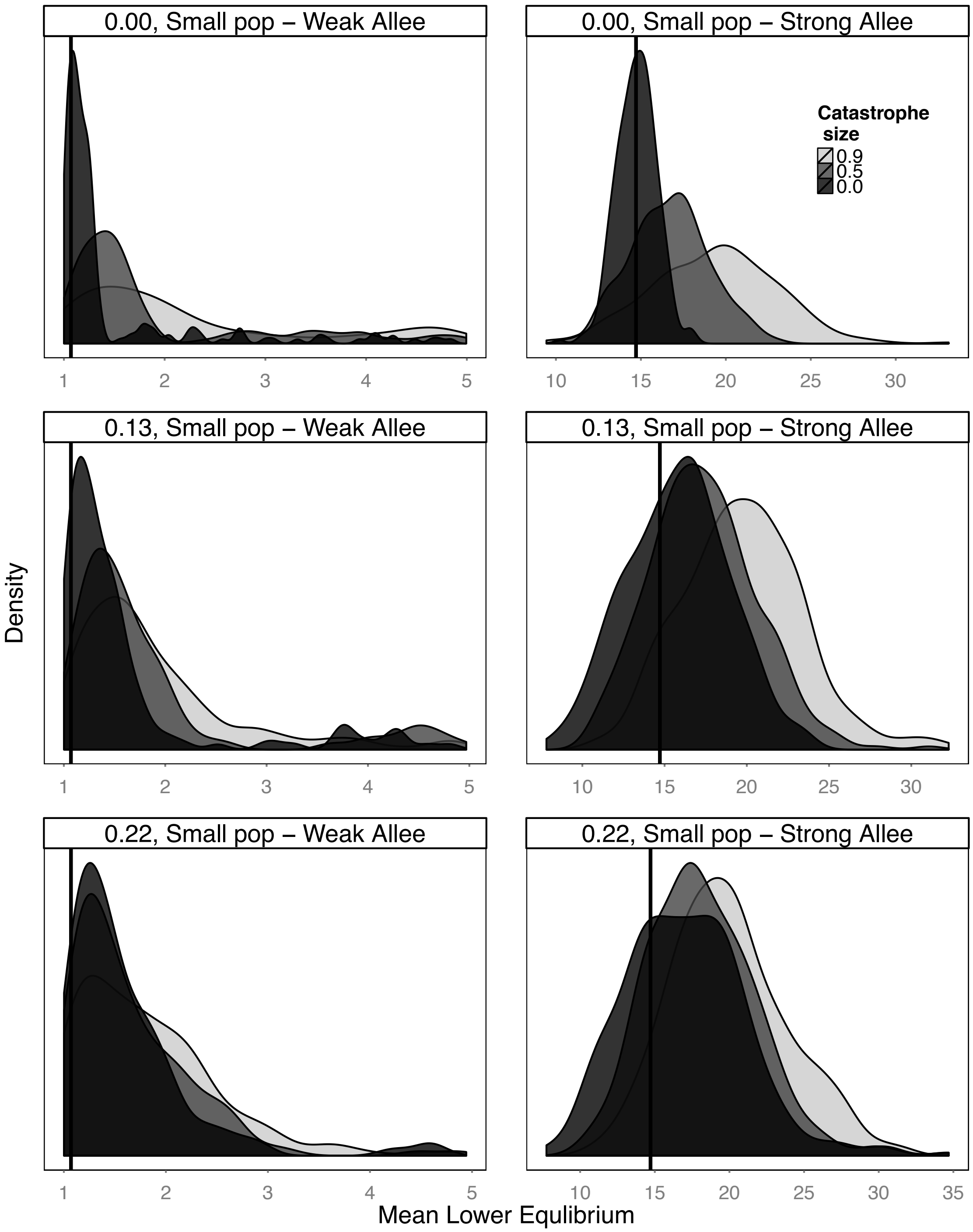
0.00, Small pop – Weak Allee
0.00, Small pop – Strong Allee
0.13, Small pop – Weak Allee
0.13, Small pop – Strong Allee
0.22, Small pop – Weak Allee
0.22, Small pop – Strong Allee
Catastrophe size
0.9
0.5
0.0
Density
Mean Lower Equlibrium
1 2 3 4 5
10 15 20 25 30 35

Figure S5

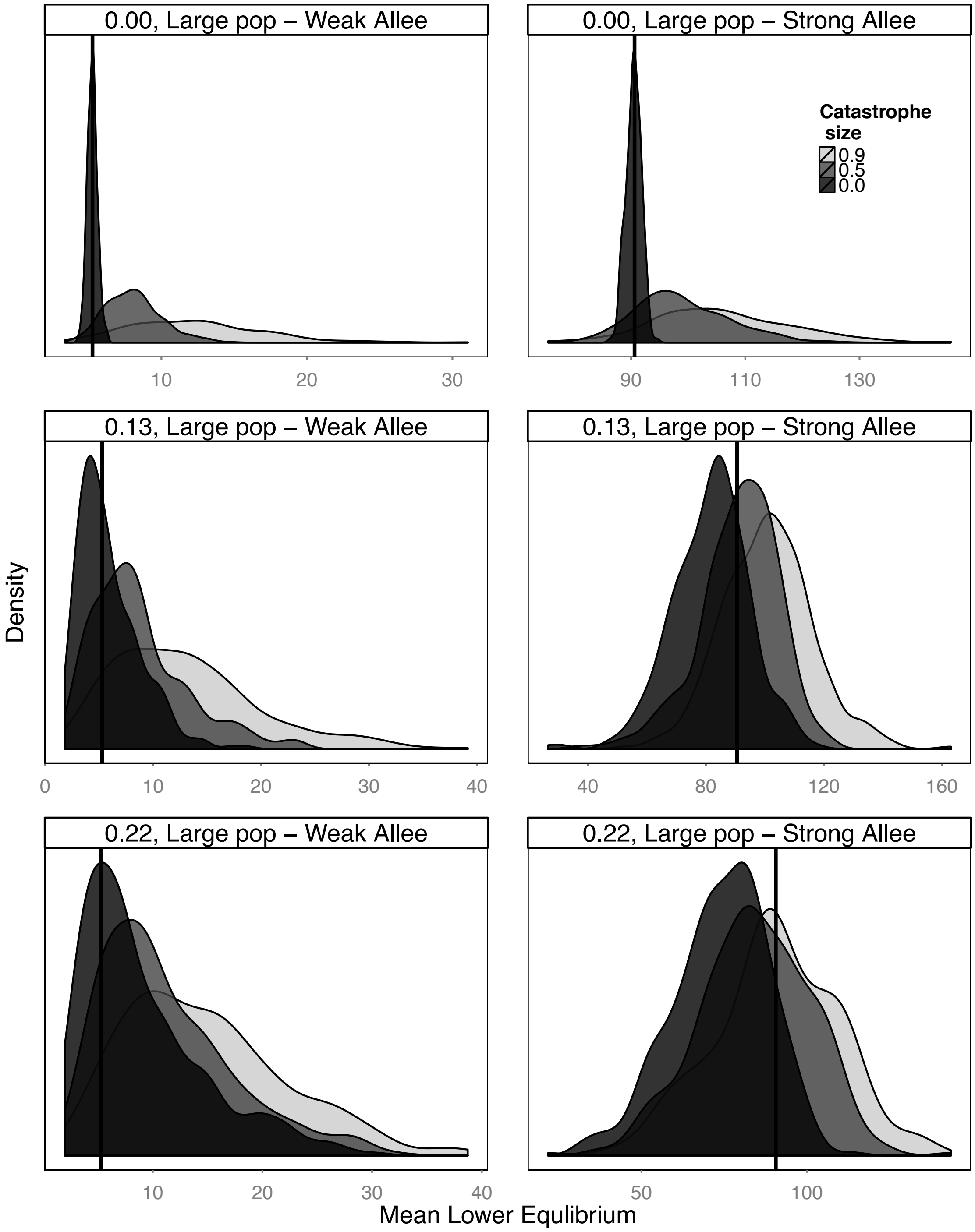
0.00, Large pop – Weak Allee
0.00, Large pop – Strong Allee
0.13, Large pop – Weak Allee
0.13, Large pop – Strong Allee
0.22, Large pop – Weak Allee
0.22, Large pop – Strong Allee
Catastrophe size
0.9
0.5
0.0
Density
Mean Lower Equlibrium

Figure S6

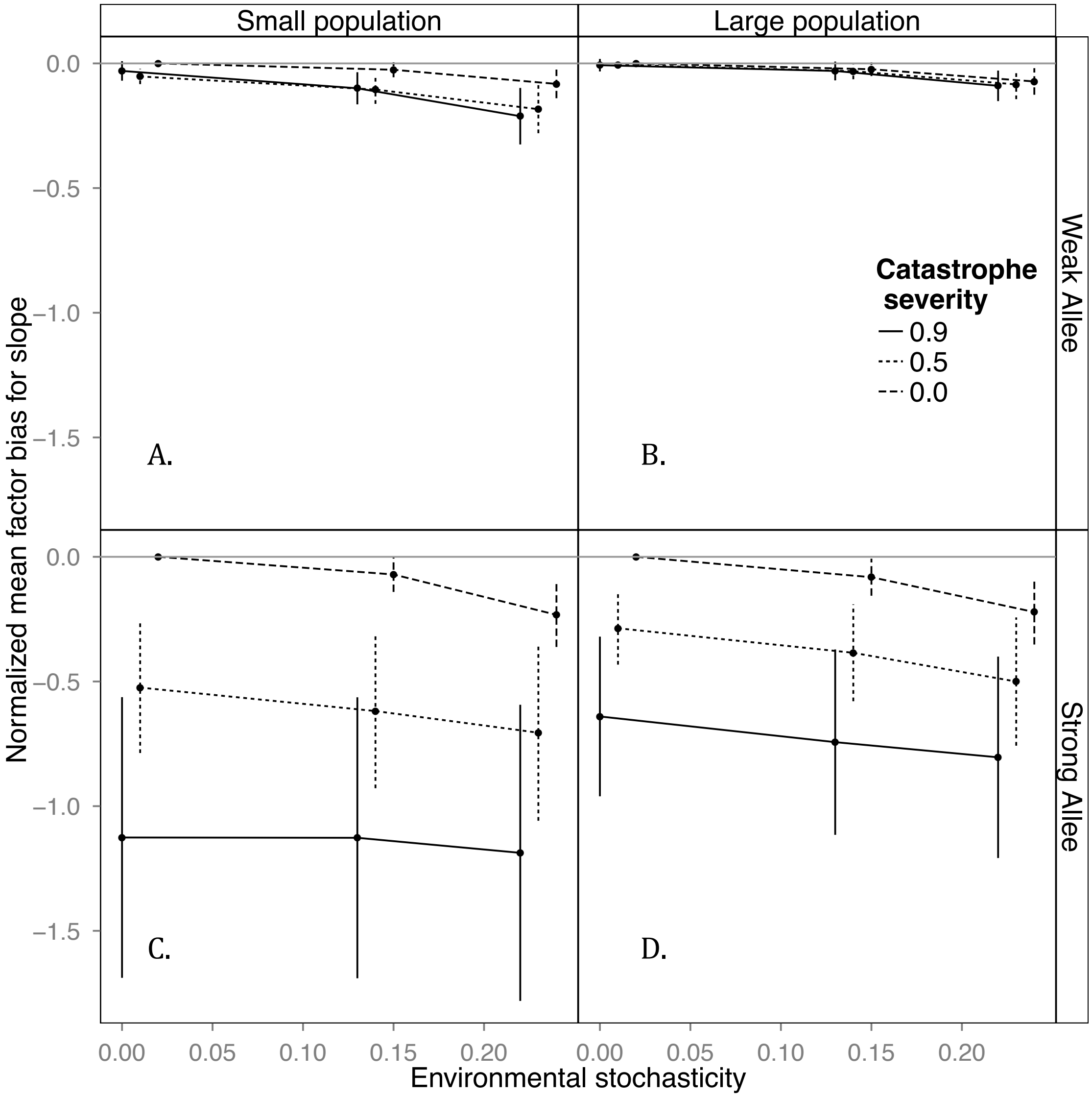

Small population
Large population
Weak Allee
Strong Allee
Normalized mean factor bias for slope
Environmental stochasticity
Catastrophe severity
0.9
0.5
0.0
A.
B.
C.
D.
0.0
−0.5
−1.0
−1.5
0.00
0.05
0.10
0.15
0.20

Figure S7

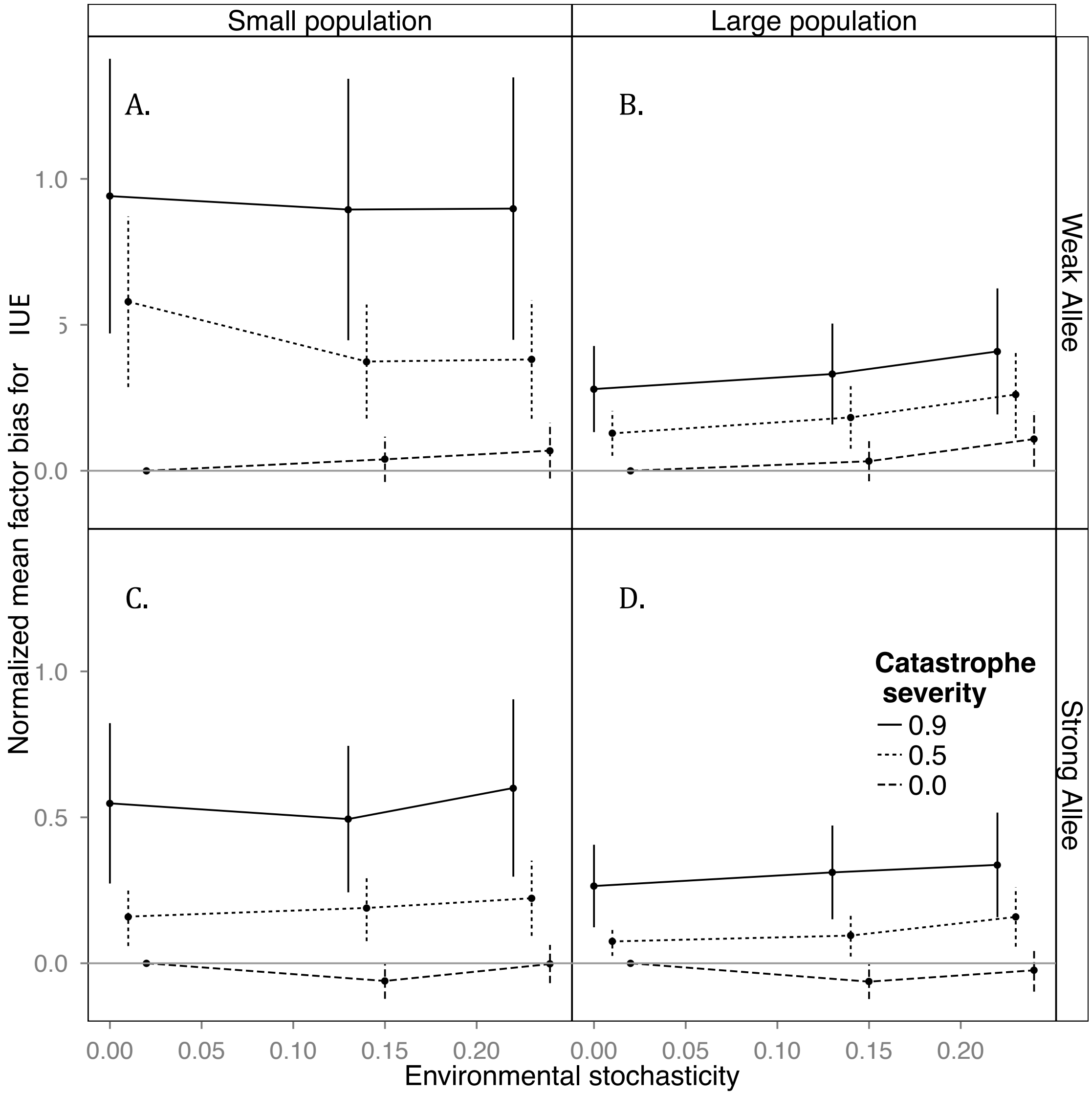

Small population
Large population
A.
B.
C.
D.
Weak Allee
Strong Allee
Normalized mean factor bias for IUE
Environmental stochasticity
Catastrophe severity
0.9
0.5
0.0
1.0
0.5
0.0
0.00
0.05
0.10
0.15
0.20
